# Pretransplant *NPM1* MRD levels predict outcome after allogeneic hematopoietic stem cell transplantation in patients with acute myeloid leukemia

**DOI:** 10.1038/bcj.2016.46

**Published:** 2016-07-29

**Authors:** S Kayser, A Benner, C Thiede, U Martens, J Huber, P Stadtherr, J W G Janssen, C Röllig, M J Uppenkamp, T Bochtler, U Hegenbart, G Ehninger, A D Ho, P Dreger, A Krämer

**Affiliations:** 1 Department of Internal Medicine V, University of Heidelberg, Heidelberg, Germany; 2 Clinical Cooperation Unit Molecular Hematology/Oncology, German Cancer Research Center (DKFZ) and Department of Internal Medicine V, University of Heidelberg, Heidelberg, Germany; 3 Division of Biostatistics, German Cancer Research Center (DKFZ), Heidelberg, Germany; 4 Department of Medicine I, University Hospital Carl-Gustav-Carus, Dresden, Germany; 5 Cancer Center Heilbronn-Franken, Heilbronn, Germany; 6 Institute of Human Genetics, University of Heidelberg, Heidelberg, Germany; 7 Department of Oncology, Hospital of Ludwigshafen, Ludwigshafen, Germany

## Abstract

The objective was to evaluate the prognostic impact of pre-transplant minimal residual disease (MRD) as determined by real-time quantitative polymerase chain reaction in 67 adult *NPM1*-mutated acute myeloid leukemia patients receiving allogeneic hematopoietic stem cell transplantation (HSCT). Twenty-eight of the 67 patients had a *FLT3*-ITD (42%). Median age at transplantation was 54.7 years, median follow-up for survival from time of allografting was 4.9 years. At transplantation, 31 patients were in first, 20 in second complete remission (CR) and 16 had refractory disease (RD). Pre-transplant *NPM1* MRD levels were measured in 39 CR patients. Overall survival (OS) for patients transplanted in CR was significantly longer as compared to patients with RD (*P*=0.004), irrespective of whether the patients were transplanted in first or second CR (*P*=0.74). There was a highly significant difference in OS after allogeneic HSCT between pre-transplant MRD-positive and MRD-negative patients (estimated 5-year OS rates of 40 vs 89% *P*=0.007). Multivariable analyses on time to relapse and OS revealed pre-transplant *NPM1* MRD levels >1% as an independent prognostic factor for poor survival after allogeneic HSCT, whereas *FLT3*-ITD had no impact. Notably, outcome of patients with pre-transplant *NPM1* MRD positivity >1% was as poor as that of patients transplanted with RD.

## Introduction

Allogeneic hematopoietic stem cell transplantation (HSCT) is considered to be the treatment strategy with the highest anti-leukemic efficacy for acute myeloid leukemia (AML) patients.^1^ Nevertheless, relapse remains the major cause of treatment failure even after allogeneic HSCT in complete remission (CR),^1, 2, 3^ suggesting that the sensitivity of morphological remission assessment is too low to allow for the detection of clinically relevant residual leukemia left behind after conventional chemotherapy. Besides achievement of morphological CR as pre-requisite for cure, the term ‘molecular remission' has been introduced for the first time in the 2003 International Working Group guidelines to refine treatment response in AML.^4^ There is increasing evidence that in AML, levels of submicroscopic amounts of leukemia cells (minimal residual disease, MRD) persisting after standard induction therapy are independently associated with increased risk of relapse and poor survival.^5^

Over the last two decades, several methods, particularly multiparameter flow cytometry (MFC) and quantitative real-time polymerase chain reaction (RT-qPCR) have been developed that enable the sensitive detection and monitoring of MRD in AML.^5^ When comparing both methods, RT-qPCR offers the highest level of sensitivity (10^−4^ to 10^−6^), depending on the AML-specific fusion gene or gene mutation measured.^5, 6^

Since frameshift mutations of the *NPM1* gene are one of the most frequent molecular abnormalities in AML and are relatively stable over time,^7, 8, 9^ they represent an ideal target for RT-qPCR MRD monitoring. To date, more than 50 different *NPM1* mutations have been reported; however, the subtypes A, B, and D comprise 90% of all variants.^10^ These three mutation subtypes have been shown to be reliable markers for MRD detection with high sensitivity.^5, 11^ The same assay can be adapted for cases with rare *NPM1* mutation variants by replacing mutation-specific primers, but case-specific RT-qPCRs need to be carefully established to avoid non-specific background amplification from the wild-type *NPM1* allele.^12^ The presence of *NPM1* MRD has consistently been shown to be associated with an adverse outcome in patients treated with chemotherapy alone.^11, 12, 13, 14, 15^ In contrast, data based on *NPM1* RT-qPCR pertaining to allogeneic HSCT are still scarce. Schnittger and colleagues reported on 252 *NPM1*-mutated AML patients, of whom 53 underwent allogeneic HSCT.^12^ However, their analyses were primarily focused on the correlation of outcome with MRD levels after chemotherapy. In a subgroup analysis they reported that a 100-fold increase of *NPM1* MRD levels in samples taken between day 61 and 365 after allogeneic HSCT was associated with a significantly inferior event-free survival. Krönke *et al.* evaluated the prognostic impact of MRD levels in 245 *NPM1*-mutated AML patients, of whom 45 patients received allogeneic HSCT.^13^ Again, *NPM1* MRD levels were a significant prognostic marker for remission duration and overall survival (OS). However, a subgroup analysis on MRD levels exclusively pertaining to allogeneic HSCT was not presented.

Pre-transplant MFC-MRD has been shown to be predictive for post-transplant outcome with high relapse rates of 60 to 70% after two or three years in MRD-positive patients as compared to only 8 to 21% in MRD-negative patients, respectively.^16, 17, 18^ The aim of this study was to evaluate the prognostic impact of pre-transplant *NPM1* MRD levels determined by RT-qPCR in correlation to clinical characteristics and genetic abnormalities assessed at initial diagnosis in a cohort of adult AML patients receiving allogeneic HSCT.

## Patients and Methods

### Patients and Treatment

Between 2005 and 2013, 238 AML patients (median age at time of allogeneic HSCT, 53.5 years; range, 17–73 years) received an allogeneic HSCT at the University of Heidelberg. Diagnosis of AML was based on standard criteria.^4^ All patients gave written informed consent in accordance with the Declaration of Helsinki. Data collection and analysis were approved by the Institutional Review Board.

Chromosome banding was performed using standard techniques, and karyotypes were described according to the International System for Human Cytogenetic Nomenclature.^19^ Based on material availability, the mutational status of *NPM1* and *FLT3*-ITD was analyzed in 208 and 215 of the 238 patients, respectively as previously described.^20, 21^ For this study, the criterion used to include patients was the presence of an *NPM1* mutation (*n*=67). In patients with a concurrent *FLT3*-ITD the allelic ratio was quantified by GeneScan-based fragment-length analysis; in cases with more than one ITD mutation, all *FLT3*-ITDs were summed-up.

All patients received intensive treatment either within clinical trials (*n*=48) or according to our local institutional standard (*n*=19). Reasons for allogeneic HSCT in first CR were: i) *FLT3*-ITD positivity (*n*=13), ii) requirement of the respective trial protocol (availability of an HLA-matched sibling donor in case of intermediate-risk patients; *n*=9), iii) secondary/therapy-related AML (*n*=5), iv) cytogenetically high-risk abnormalities (*n*=2), and v) raising *NPM1* MRD levels (*n*=2). Induction regimens included intensive chemotherapy according to the ‘7+3' scheme (cytarabine (Ara-C) 100 mg/m^2^, d1-7 plus daunorubicin 60 mg/m^2^, d3-5) or Ara-C 1 g/m^2^ bid, d1,3,5,7 plus mitoxantrone (mito) 10 mg/m^2^, d1-3 and pegfilgrastim 6 mg s.c., d10. Consolidation therapy consisted of age-adapted high-dose Ara-C (3 g/m^2^, bid, d1,3,5 for patients age ⩽60 years and 1 g/m^2^, bid, d1,3,5 for patients >60 years) or age-adapted mito (10 mg/m^2^, d4-6 for patients ⩽60 years and 10 mg/m^2^, d1+2 and pegfilgrastim 6 mg s.c., d8 for patients >60 years) plus age-adapted Ara-C (1 g/m^2^, bid, d1-6 for patients ⩽60 years and Ara-C 500 mg/m^2^ bid, d1,3,5, for patients >60 years) or mito 10 mg/m^2^, d4-6 with amsacrine 100 mg/m^2^, d1-5 and Ara-C 1 g/m^2^, bid, d1-5. In refractory or relapsed patients either mito and high-dose Ara-C; mito 10 mg/m^2^, d1-5 and etoposide 100 mg/m^2^, d1-5 (NOVE);^22^ fludarabine 30 mg/m^2^ plus high-dose Ara-C 2 g/m^2^ and amsacrine 100 mg/m^2^ for four days (FLAMSA);^23^ clofarabine 40 mg/m^2^, d2-6 and Ara-C 1 g/m^2^, d1-5^24^ or fludarabine/Ara-C/granulocyte colony-stimulating factor/idarubicin (Flag-IDA)^25^ have been used as salvage therapy.

In total 67 patients with *NPM1*-mutated AML were included into the analysis and data pertaining to these patients were collected from electronic patient records with follow-up until July 27, 2015.

### Detection of MRD

Collection of bone marrow (BM) samples for MRD analysis was recommended at diagnosis, during aplasia within induction therapy, after each treatment cycle and every three months after completion of therapy. RT-qPCR analysis was performed at diagnosis and follow-up on cDNA obtained from BM (*n*=406) specimens as described previously.^11, 13, 26^ MRD levels were expressed as a ratio of the *NPM1* mutation normalized to the housekeeping gene *ABL1* to adjust for variations in mRNA quality and efficiencies of cDNA synthesis. To increase external validity and to be consistent with a previous report indicating a poor survival of AML patients after chemotherapy^26^ we used a cut-point of 1% (less than 100 copies of mutated *NPM1*/10^4^ ABL1 copies) to define MRD-negativity prior to allogeneic HSCT. The sensitivity level was 10^−5^ to 10^−6^.

### Statistical analyses

CR and survival endpoints such as OS, relapse-free survival (RFS), time to relapse (TTR) and time to non-relapse mortality (NRM) were defined as recommended.^27^ All event times were measured from date of allogeneic HSCT. For OS all 67 patients were considered. The analysis of RFS and competing risk analysis of TTR vs NRM was restricted to *NPM1*-mutated patients transplanted in CR (*n*=51). Cytogenetic categorization into favorable-, intermediate- and adverse-risk groups followed recommended criteria.^28^ Pairwise comparisons between patient characteristics (covariates) were performed by the Mann-Whitney U test for continuous variables and by Fisher's exact test for categorical variables. The follow-up distribution was computed using the reverse Kaplan-Meier estimate.^29^ The Kaplan-Meier method was used to estimate the distribution of RFS and OS.^30^ Confidence interval (CI) estimation for survival curves was based on the cumulative hazard function using Greenwood's formula for variance estimation. Logrank tests were employed to compare survival curves between groups. Cumulative incidence of relapse (CIR) and cumulative incidence of death in remission (CID) were computed using the Aalen-Johansen estimator^31^ and included only patients attaining CR. A Cox proportional hazards regression model was used to identify prognostic variables for OS and RFS.^32^ For competing risks analyses a cause-specific Cox model was used. The following variables were included in the Cox models: achievement of CR prior to allogeneic HSCT in combination with pre-transplant MRD status (for OS only), age, percentage of BM blasts, lactate dehydrogenase (LDH), *FLT3*-ITD and MRD positivity prior to allogeneic HSCT (for TTR only). All statistical analyses were performed with the statistical software environment R, version 3.1.3, using the R packages rms, version 4.3-1, prodlim, version 1.5.1, coxphf, version 1.11, and survival, version 2.38-1.^33^

## Results

### Pretreatment characteristics and factors pertaining to allogeneic HSCT

Genetic risk category was intermediate in 62 of the 67 *NPM1*-mutated patients based on revised Medical Research Council/National Cancer Research Institute criteria^28^ and most of them (*n*=52) had a normal karyotype. Three patients belonged to the adverse risk category^28^ and two patients had no evaluable metaphases. The *FLT3* status was measured in all *NPM1*-mutated patients. A *FLT3*-ITD was present in 28 of the 67 patients (42%); the allelic ratio could be measured in 24 (86%) of the *FLT3*-ITD patients, with a median of 0.58 (range 0.03-14.3). Source of donor was matched-related in 20, matched-unrelated in 45 and haplo-identical in 2 of the 67 patients, respectively. The majority of patients (*n*=61) received reduced-intensity conditioning (RIC), consisting of either melphalan/fludarabine (*n*=27),^34^ treosulfan/fludarabine (*n*=11),^35^ busulfan/fludarabine (*n*=4), busulfan/fludarabine plus amsacrine/Ara-C (*n*=6), fludarabine/TBI, 2–8 Gy (*n*=12) and cyclophosphamide/TBI, 4 Gy (*n*=1).^36, 37, 38, 39^ Disease status at allogeneic HSCT was CR1 in 31, CR2 in 20 and refractory in 16 patients, without significant differences for other baseline characteristics between the three groups (Table 1). Similarly, there was no difference between MRD positive (*n*=22) and MRD negative (*n*=17) patients allografted in CR (Table 2).

### Evaluation of MRD

Of the 51 *NPM1*-mutated patients who were in CR at allogeneic HSCT, pre-transplant MRD was assessed in 39 patients. MRD was measured in BM within one month prior to allogeneic HSCT in 28 (72%) and within two months in 11 of the 39 (28%) patients, respectively. Twenty-two of the 39 (56%) patients were MRD-positive and 17 (44%) MRD-negative. MRD was not measured in RD patients.

### Survival analysis

The estimated median follow-up for survival of the 67 *NPM1*-mutated patients was 4.9 years (95%-CI, 3.8 to 6.2 years); the estimated 5-year OS rate was 57% (95%-CI, 44% to 68%). For patients in first and second CR the estimated 5-year OS rate was 60% (95%-CI, 40% to 75%) and 68% (95%-CI, 41% to 84%), respectively. Patients with refractory disease (RD) had an estimated 5-year OS rate of 38% (95%-CI, 15% to 60%). OS for patients transplanted in CR was significantly longer as compared to patients with RD (*P*=0.004), irrespective of whether the patients were transplanted in first or second CR (*P*=0.74). The estimated CIR and CID at 5 years for patients transplanted in first as compared to second CR were 32% (95%-CI, 16% to 49%) and 11% (95%-CI, 0% to 23%) for first CR, and 34% (95%-CI, 11% to 56%) and 5% (95%-CI, 0% to 15%) for second CR patients, respectively.

**Figure 1:**
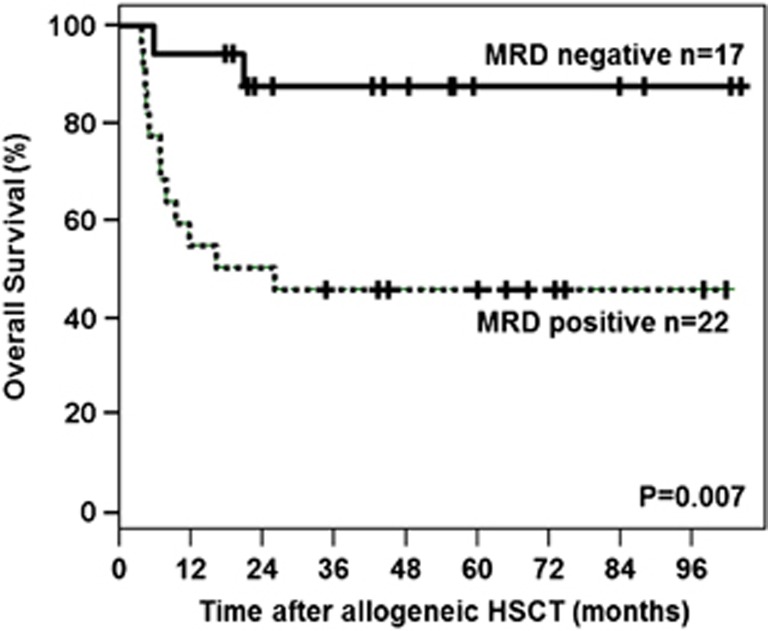
Kaplan-Meier plot illustrating the impact of pre-transplant *NPM1* minimal residual disease on overall survival of patients with complete remission.

There was no difference in outcome for CR patients with (*n*=39) or without (*n*=12) MRD measurement (*P*=0.84 for OS). Yet, there was a highly significant difference in OS after allogeneic HSCT between pre-transplant MRD-positive and MRD-negative patients, with estimated 5-year OS rates of 40 vs 89%, respectively (*P*=0.007; Figure 1).

**Figure 2:**
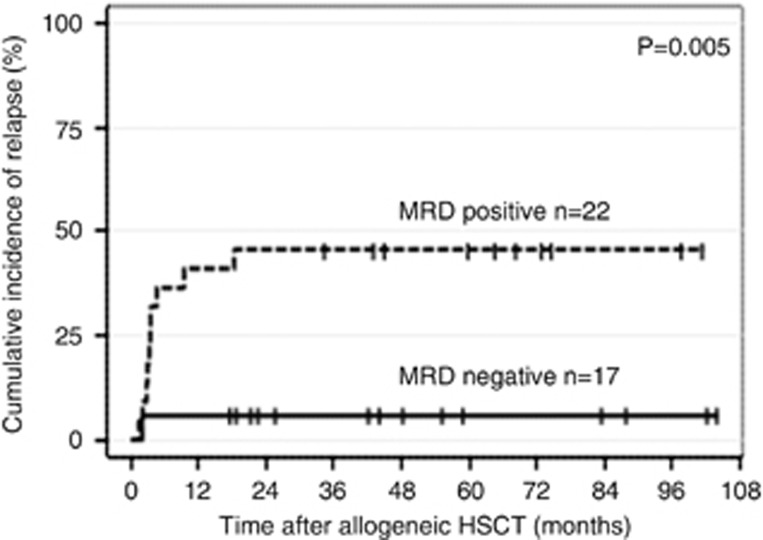
Cumulative incidence of relapse according to minimal residual disease status in *NPM1*-mutated AML patients transplanted in complete remission.

Considering MRD-positive patients (*n*=22) the estimated CIR at 5 years was 46% (95%-CI, 25% to 66%) as compared to 6% (95%-CI, 0% to 17%) for MRD-negative patients (*n*=17; Figure 2). CID at 5 years was comparable between the two groups (MRD-positive: 9% 95%-CI, 6% to 21% MRD-negative: 7% 95%-CI, 0% to 19%).

In total, 16 relapses occurred in patients transplanted in CR: nine of 32 (28%) *NPM1*-mutated/*FLT3*-ITD negative patients and seven of 19 (37%) *NPM1-*mutated/*FLT3*-ITD positive patients relapsed. With regard to the pre-transplant MRD status of *NPM1*-mutated/*FLT3*-ITD negative patients, seven of 11 (64%) MRD-positive patients relapsed, whereas none of 13 MRD-negative patients experienced disease recurrence so far. With respect to *NPM1*-mutated/*FLT3*-ITD positive patients three of 11 (27%) MRD-positive patients and one of four MRD-negative patients relapsed after allogeneic HSCT. The latter patient experienced MRD negativity for only three months prior to allogeneic HSCT and relapsed with the same *NPM1* subtype already two months after myeloablative allogeneic HSCT.

Based on previous reports showing a differential impact of *FLT3*-ITD according to its allelic ratio^40, 41, 42^ we performed additional exploratory subgroup analyses. Considering a dichotomized allelic ratio with a cutoff of 0.5^40, 41, 42^ no significant prognostic impact was evident for OS (*P*=0.36), albeit based on a small subgroup analysis (*n*=24) only.

In a multivariable cause-specific Cox model on TTR, the hazard ratio for pre-transplant MRD was 9.0 (95%-CI: 1.1–75.9; *P*=0.04) in the subset of patients in CR, whereas the *FLT3*-ITD status measured at diagnosis had no impact (*P*=0.92; Table 3a). The same held true in a Cox model on OS (Table 3b). Additional significant and trendwise important variables were achievement of CR in combination with pre-transplant MRD status (on OS) as well as LDH value (on TTR and OS) whereas age at the time of allogeneic HSCT and percentage of BM blasts at diagnosis had no impact (Tables 3a and 3b). Again, there was no significant difference in outcome for patients in first or second CR (data not shown). A model on NRM was not performed due to a very low event number for this endpoint (3 out of 39 patients).

**Figure 3:**
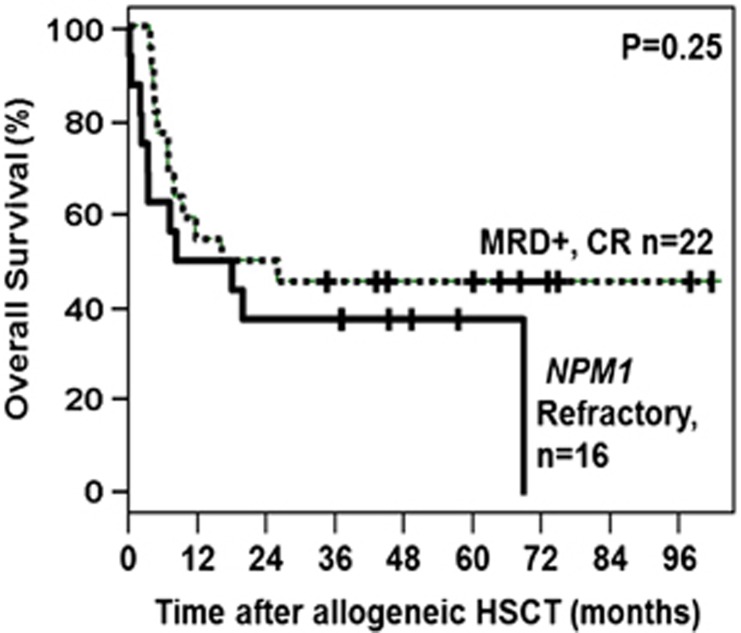
Overall survival of pre-transplant *NPM1* minimal residual disease positive patients as compared to *NPM1*-mutated patients transplanted with refractory disease.

The 5-year OS rate of patients receiving an allogeneic HSCT in refractory disease was 38% and comparable to that observed in pre-transplant MRD-positive patients (40% *P*=0.42; Figure 3).

## Discussion

The focus of our study was to assess the prognostic impact of *NPM1* MRD prior to allogeneic HSCT on TTR and OS. Albeit performed on a limited number of patients, in our cohort of *NPM1*-mutated patients pre-transplant *NPM1* MRD positivity was a significant predictor of poor outcome after allogeneic HSCT independent from other variables, including *FLT3*-ITD status, BM blast count at diagnosis and age which adds to recently published data.^12, 13^ Of note, prognosis of MRD-positive patients was not better than that of patients transplanted in RD.

Outcome data of *NPM1*-mutated patients without *FLT3*-ITD transplanted in first as compared to second CR are still scarce. At least until recently, those patients were not transplanted in first CR. Regarding our patient cohort our results are in line with the data published by Alan Burnett *et al.* who reported on a 5 years CIR in *NPM1-*mutated/*FLT3*-ITD negative patients of 39% and a 5-year survival from CR of 68% when the patients who received salvage treatment were considered.^43^

In several studies it has been shown that the prognostic impact of *NPM1* should be interpreted in the context of a cooperating *FLT3*-ITD mutation, which is present in approximately 45% of this patient population with normal karyotype.^21, 44, 45^ In particular, in younger adult *NPM1*-mutated patients with high *FLT3*-ITD allelic ratio (⩾0.5)^40, 41, 42^ the favorable prognostic effect of *NPM1* is mitigated or even abolished as compared to patients with a low allelic ratio.^21, 41, 42^ Nevertheless, in none of these publications the relative impact of the *FLT3*-ITD allelic ratio on the background of *NPM1* MRD has been evaluated. In our study MRD positivity prior to allogeneic HSCT turned out to be a stronger predictor of relapse than *FLT3*-ITD at initial diagnosis, which adds to recently published data reporting an increased relapse risk associated with raising *NPM1* MRD levels of >1% despite of having achieved CR after completion of chemotherapy.^26^ In line with our data, in the paper by Shayegi *et al. FLT3*-ITD had no further prognostic information after conventional chemotherapy and autologous HSCT if *NPM1* MRD level was considered.^26^ Moreover, in the recently published paper by Ivey *et al.* the presence of *NPM1* MRD was the only significant prognostic factor in multivariable analysis for relapse and death, whereas the presence of a *FLT3*-ITD did not provide additional prognostic information.^15^

Data regarding MRD and specific *FLT3*-ITD characteristics, such as the allelic ratio and ITD insertion site in the *FLT3* gene, are still scarce. In line with our data, Ivey *et al.* could not find a difference in the allelic burden according to *NPM1* MRD level.^15^

Considering a cutoff value of 0.5,^40, 41, 42^ the *FLT3*-ITD ratio had no impact on OS in our analyses, albeit based on a small subgroup analysis only. Based on availability of material, we could neither address the impact of ITD insertion site nor evaluate the *FLT3*-ITD mutational status at the time-point of allogeneic HSCT. Therefore, one possibility why *FLT3*-ITD seemed not to be associated with outcome after allogeneic HSCT could be that the clone present at the time of allogeneic HSCT was ITD negative.

RFS and OS for *NPM1* MRD-positive patients transplanted in CR was identical in our cohort (data not shown), suggesting that once relapse occurred, further treatment with tapering of immunosuppression, chemotherapy or even a second allogeneic HSCT had no major impact on survival. A major benefit of allogeneic HSCT performed in CR was only present in patients who had *NPM1* MRD levels below 1% prior to allogeneic HSCT. Hence, current practice to recommend allogeneic HSCT without considering MRD levels has to be called into question. Currently it is unclear, if MRD-positive patients would benefit from additional cycles of pre-transplant high-dose Ara-C or other intensification. Several retrospective studies have suggested that standard Ara-C-based consolidation chemotherapy before allogeneic HSCT for AML patients of all risk-groups in first CR does not improve post-transplant outcomes.^46, 47, 48, 49^ However, in none of these trials information on MRD was available, and it is unknown whether the subset of MRD-positive patients would benefit from additional post-remission therapy prior to allogeneic HSCT.

Another strategy to overcome MRD could be increasing conditioning intensity. Several retrospective analyses as well as one prospective study reported comparable outcomes after myeloablative vs RIC in AML,^50, 51, 52, 53^ whereas current data by the Blood and Marrow Transplant Clinical Trials Network suggest a beneficial impact of myeloablative over RIC in patients with myelodysplastic syndromes or AML.^54^ Yet again, in none of these studies data on MRD were available. Since most of our patients have received RIC, the impact of myeloablative conditioning on MRD levels should be addressed further.

Numerous studies have convincingly demonstrated that MRD positivity before allogeneic HSCT, determined by MFC, is independently associated with a significantly increased risk of relapse and inferior survival.^16, 17, 18, 55, 56, 57^ Assuming that a further reduction of MRD levels optimizes outcome after allogeneic HSCT, this relationship would justify risk-stratified treatment allocation, including the use of additional pre-transplant chemotherapy. However, as MRD might simply reflect reduced sensitivity of leukemia cells to chemotherapy, the presence of residual disease might only mark those patients who are unlikely to be cured with subsequent similar-type therapies, even if disease levels are brought temporarily below the level of detection. Therefore, another approach could be pre-emptive immunotherapy in MRD-positive patients,^58^ which has successfully been demonstrated in childhood AML with mixed chimerism after allogeneic HSCT,^59^ or by post-transplant application of demethylating agents, such as azacitidine, to prevent imminent relapse in MRD-positive patients.^60^

In summary, our data provide clinically relevant information that may allow to improve post-transplant outcome in MRD-positive patients with *NPM1-*mutated AML. In addition, pre-transplant *NPM1* MRD levels seem to outperform the prognostic information provided by *FLT3*-ITD with regard to outcome after allogeneic HSCT. Nonetheless, this observation warrants confirmation in further studies.

## Figures and Tables

**Table 1:** Patient characteristics by disease status prior to allogeneic hematopoietic stem cell transplantation in *NPM1*-mutated patients

*Remission status prior to allogeneic HSCT*	*1st CR (*n*=31)*		*2nd CR (*n*=20)*		*RD (*n*=16)*		P*-value*
	No.	%	No.	%	No.	%	
Median age (at time of SCT; years)	57.1		48.0		57.0		
Range	21.0–70.8		29.2–70.1		43.3–63.4		0.13
							
*Gender*							
Male	13	41.9	14	70	7	43.8	0.13
Female	18	58.1	6	30	9	56.2	
							
*Type of AML*							
*de novo*	26	83.9	19	95	14	87.5	0.51
secondary	2	6.5	1	5	2	12.5	
therapy-related	3	9.7	-	-	-	-	
							
*Hemoglobin, g/dl*							
Median	9.6		8.7		10.4		0.22
Range	6.2–12.2		4.6–13.5		5.8–12.5		
No. missing	-		-		-		
							
*WBC, x10*^*9*^*/l*							
Median	52.3		46.2		28.3		0.77
Range	1.4–348.5		4.0–160.4		2.1–290		
No. missing	-		-		-		
							
*Platelet count, x10*^*9*^*/l*							
Median	77		83		103.5		0.63
Range	4–369		28–379		23–242		
No. missing	-		-		-		
							
*LDH value, U/l*							
Median	540		539		446.5		0.67
Range	144–2741		128–2083		139–2573		
No. missing	-		1		-		
							
*Percentage of BM blasts*							
Median	80		76		80		0.82
Range	25–100		27–95		47–90		
No. missing	-		-		-		
							
*Cytogenetic risk category*^a^							
intermediate	29	93.5	20	100	13	92.9	0.42
high	2	6.5	-	-	1	7.1	
No. missing	-		-		2		
							
*FLT3*-ITD mutated	13	41.9	6	30	9	56.2	0.29
No. missing	-		-		-		
							
*Donor type*							
MUD	18	58.1	14	70	13	81.2	0.10
MRD	13	41.9	4	20	3	18.8	
Haplo	-		2	10	-		

Abbreviations: AML, acute myeloid leukemia; BM, bone marrow; CR, complete remission; HSCT, hematopoietic stem cell transplantation; ITD, internal tandem duplication; LDH, lactate dehydrogenase; RD, refractory disease; WBC, white blood count.

a According to reference.^28^

a Percentages may not add to 100 because of rounding.

**Table 2:** Comparison of clinical and laboratory findings according to minimal residual disease status prior to allogeneic hematopoietic stem cell transplantation in *NPM1*-mutated patients in complete remission

*MRD status prior to allogeneic HSCT*	*MRD-positive (*n*=22)*		*MRD-negative (*n*=17)*		P*-value*
	No.	%	No.	%	
Median age (at time of SCT; years)	51.1		52.5		0.49
Range	21.0–70.1		29.2–70.8		
					
*Gender*					
Male	13	59.1	7	41.2	0.34
Female	9	40.9	10	58.8	
					
*Type of AML*					
*de novo*	20	90.9	14	82.3	0.78
secondary	1	4.5	2	11.8	
therapy-related	1	4.5	1	5.9	
					
*Hemoglobin, g/dL*					
Median	9.2		9.7		0.54
Range	6.2–13.3		4.6–12.7		
No. missing	-		-		
					
*WBC, x10*^*9*^*/L*					
Median	62.0		31.6		0.07
Range	1.9–348.5		1.4–153.0		
No. missing	-		-		
					
*Platelet count, x10*^*9*^*/L*					
Median	85		77		0.98
Range	4–210		14–369		
No. missing	-		-		
					
*LDH value, U/L*					
Median	667		420		0.06
Range	203–2449		144–1112		
No. missing	-		-		
					
*Percentage of BM blasts*					
Median	82.5		75		0.31
Range	41–100		40–96		
No. missing	-		-		
					
*Cytogenetic risk category*^a^					
intermediate	21	95.5	16	94.1	1.00
high	1	4.5	1	5.9	
No. missing	-		-		
					
*FLT3*-ITD mutated	11	50	4	23.5	0.11
No. missing	-		-		
					
*Donor type*					
MUD	15	68.2	9	52.9	0.16
MRD	5	22.7	8	47.1	
Haplo	2	9.1	-		
					
*Achievement of CR*					
CR1	14	63.6	11	64.7	1.00
CR2	8	36.4	6	35.3	

Abbreviations: AML, acute myeloid leukemia; BM, bone marrow; CR, complete remission; HSCT, hematopoietic stem cell transplantation; ITD, internal tandem duplication; LDH, lactate dehydrogenase; WBC, white blood count.

a According to reference. Percentages may not add to 100 because of rounding.

**Table 3a:** Multivariable cause-specific Cox model on time to relapse in *NPM1*-mutated patients with acute myeloid leukemia allografted in complete remission (*n*=39)

	*HR (95%-CI)*	P*-value*
*FLT3*-ITD	0.91 (0.16–5.26)	0.92
Log_10_(LDH) (at diagnosis)	7.87 (0.93–66.4)	0.06
Percentage of BM blasts at diagnosis; 10% increase	1.05 (0.67–1.66)	0.82
Age at the time of allogeneic HSCT; 10 years increase	1.67 (0.82–3.39)	0.16
Pre-transplant MRD positivity	9.03 (1.07–75.9)	0.04

Abbreviations: BM, bone marrow; CI, confidence interval; HR, hazard ratio; HSCT, hematopoietic stem cell transplantation; ITD, internal tandem duplication; LDH, lactate dehydrogenase; MRD, minimal residual disease.

**Table 3b:** Multivariable Cox model on overall survival in 55 *NPM1*-mutated patients with acute myeloid leukemia (including patients in complete remission with measurement of minimal residual disease and patients with refractory disease)

	*HR (95%-CI)*	P*-value*
*FLT3*-ITD	0.58 (0.17–1.93)	0.37
Log_10_(LDH) (at diagnosis)	6.65 (1.25–35.3)	0.03
Percentage of BM blasts at diagnosis; 10% increase	1.24 (0.90–1.72)	0.19
Age at the time of allogeneic HSCT; 10 years increase	1.56 (0.92–2.64)	0.10
		
*Achievement of CR and pre-transplant MRD status (reference RD)*		
CR, MRD-positive	0.70 (0.29–1.70)	0.02
CR, MRD-negative	0.11 (0.02–0.50)	

Abbreviations: BM, bone marrow; CI, confidence interval; CR, complete remission; HR, hazard ratio; HSCT, hematopoietic stem cell transplantation; ITD, internal tandem duplication; LDH, lactate dehydrogenase; MRD, minimal residual disease; RD, refractory disease.
